# Knockdown of Expression of Cdk5 or p35 (a Cdk5 Activator) Results in Podocyte Apoptosis

**DOI:** 10.1371/journal.pone.0160252

**Published:** 2016-08-01

**Authors:** Ya-Li Zheng, Xia Zhang, Hai-Xia Fu, Mei Guo, Varsha Shukla, Niranjana D. Amin, Jing E, Li Bao, Hong-Yan Luo, Bo Li, Xiao-Hua Lu, Yong-Cai Gao

**Affiliations:** 1 Department of Nephrology, Ningxia People’s Hospital (First Affiliated Hospital of Medical College, Northwest University for Nationalities), Yinchuan, Ningxia Province, 750002, China; 2 Center of Blood Purification, Central Hospital of Rizhao City, Rizhao, Shangdong Province, 276800, China; 3 Laboratory of Neurochemistry, National Institute of Neurological Disorders and Stroke (NINDS), Bethesda, MD, 20814, United States of America; 4 Zhongshan-Xuhui hospital affiliated Fudan University, Shanghai, 200031, China; McGill University Department of Neurology and Neurosurgery, CANADA

## Abstract

Podocytes are terminally differentiated glomerular epithelial cells. Podocyte loss has been found in many renal diseases. Cdk5 is a cyclin-dependent protein kinase which is predominantly regulated by p35. To study the role of Cdk5/p35 in podocyte survival, we first applied western blotting (WB) analysis to confirm the time-course expression of Cdk5 and p35 during kidney development and in cultured immortalized mouse podocytes. We also demonstrated that p35 plays an important role in promoting podocyte differentiation by overexpression of p35 in podocytes. To deregulate the expression of Cdk5 or p35 in mouse podocytes, we used RNAi and analyzed cell function and apoptosis assaying for podocyte specific marker Wilms Tumor 1 (WT1) and cleaved caspase 3, respectively. We also counted viable cells using cell counting kit-8. We found that depletion of Cdk5 causes decreased expression of WT1 and apoptosis. It is noteworthy, however, that downregulation of p35 reduced Cdk5 activity, but had no effect on cleaved caspase 3 expression. It did, however, reduce expression of WT1, a transcription factor, and produced podocyte dysmorphism. On the other hand increased apoptosis could be detected in p35-deregulated podocytes using the TUNEL analysis and immunofluorescent staining with cleaved caspase3 antibody. Viability of podocytes was decreased in both Cdk5 and p35 knockdown cells. Knocking down Cdk5 or p35 gene by RNAi does not affect the cycline I expression, another Cdk5 activator in podocyes. We conclude that Cdk5 and p35 play a crucial role in maintaining podocyte differentiation and survival, and suggest these proteins as targets for therapeutic intervention in podocyte-damaged kidney diseases.

## Introduction

Cyclin-dependent kinase 5 (Cdk5), a serine/threonine protein kinase, which forms active complexes with p35 or p39 is essential to neural development and function. Cdk5 activity, however, when deregulated, contributes to neurodegeneration as in Alzheimers disease as well as pancreatic dysfunction as in the pathogenesis of Type 2 diabetes mellitus[1–4].It has also been reported that expression of Cdk5 and p35 are important in development and function of kidney podocytes [5].

Podocytes are specialized, terminally differentiated visceral epithelial cells that reside on the glomerular basement membrane outside the glomerular capillaries[6]. Podocytes, fenestrated endothelium, and the intervening glomerular basement membrane (GBM) make up the glomerular filtration barrier [6–7]. The integration of this structure is essential in the maintenance of glomerular filtration. There is compelling evidence that podocyte damage and loss contribute to the initiation of glomerulosclerosis and progression of chronic renal diseases [6, 8–11].

Both hypoactivity and hyperactivity of Cdk5/p35 causes pathological damage [12–14]. For example, knockout of Cdk5 in mice results in more than 60% fetal death *in utero*, while newborns become weak and die within 12h after birth [15]. These results demonstrated a critical role for Cdk5 in corticogenesis and in the normal cytoskeletal architecture of the developing neurons. Another study by Chae et al showed that mice lacking p35, display severe cortical lamination defects and suffer from sporadic adult lethality and seizures [16]. Studies of the role of cdk5/p35 in podocytes, however, are limited. Only one study showed that lack of p35 expression can easily cause the podocyte apoptosis under some special conditions such as exposure to UV-C irradiation, serum depletion, or puromycin aminonucleoside treatment [5]. Here, results of experiments to determine the role of Cdk5/p35 expression in podocytes show that both proteins and the activity of Cdk5/p35 are essential for podocyte function and survival.

## Materials and Methods

### Animals

Pregnant C57BL/6J mice were obtained from Jackson Laboratory (Bar Harbor, ME, USA). The mice welfare and steps: mice were housed and bred in the animal facility of National Institute of Neurological Disorders and Stroke (NINDS) (protocol is ASP: 1231–11) in National Institutes of Health (NIH) in the accordance with the U.S. National Institutes of Health Guide for Care and Use of Laboratory Animals. Totally, 19 mice were used for the study (including four pregnant mothers; five babies for p2 experiments; and ten adults for adult’s experiments). Mice were group housed with a 12-hr light/dark cycle and had *ad libitum* access to food and water. Mice were anesthetized with CO_2_+20%O_2_ at the time point following time course dependent manner (the time point of sacrifice was at E14, E18, E22, p2 and adult), and then harvested kidneys for the further experiments. Five adult’s kidneys were used for the glomerular isolation. The rest of them were used to harvest the kidneys for renal cortical protein analysis,then submitted to the western blot. Mice kidney harvest was carried out in the animal facility of NINDDK (protocol is K058-KDB-10) by two very skillful researcher and technician who have very good training on animal programs in NIH and have been worked in animal experiment field for many years.

### Antibodies and reagents

Cdk5(C-8), p35(C-19) polyclonal antibodies, Cdk5 (J-3) monoclonal antibody (1:1000/1:50), Cyclin I polyclonal antibody (1:500) and WT1 monoclonal antibody (1:2000) were obtained from SantaCruz. Cleaved caspase-3(Asp175) polyclonal antibody (1:2000/1:200) was purchased from Cell Signaling. Anti-tubulin monoclonal antibody (1:2500) and anti-β-actin monoclonal antibody (1:2000) were purchased from Sigma. Tunel-TMR kit was obtained from Roche. Cell counting kit-8 (CCK-8) was got from Sigma Aldrich.

### Cell culture

Immortalized mouse podocytes (provided by Dr. Shankland’s group, University of Washington Seattle, Seattle, USA) were cultured according to a published procedure Griffin et al [17]. Briefly, the cells were grown in RPMI-1640 medium supplemented with 10% FBS, 2mmol/L glutamine, 10mmol/L HEPES, 1mmol/L sodium pyruvate, 100 U/ml penicillin and 0.1 mg/ml streptomycin. To induce proliferation, cells were grown on the collagen I coated plates (Becton Dickinson Labware) with the addition of 10 U/ml recombinant mouse γ-interferon to the culture medium, and incubated at 33°C (growth permissive conditions). To induce differentiation, cells were grown in the same culture medium without γ-interferon and incubated at 37°C (growth restrictive conditions).Cells cultured for 10–14 days were used for the experiments. Cortical neurons and HEK293 cells were cultured as previously described [18].

### Transfection of short interfering RNA (siRNA) and infection of p35

Cdk5 siRNA (sc-29263), p35 siRNA (sc-36154), and control siRNA (sc-37007) were ordered from Santa Cruz Inc and delivered to podocytes using Pepmute siRNA transfection reagent (SignaGen Laboratories), according to the manufacturer's instructions. Briefly, podocytes were seeded in a 6-well plate and cultured for 7 days at about 50–60% confluence. For transfection, 5μl siRNA were diluted in 100μl of 1x siRNA transfection buffer (SignaGen Laboratories) in a final concentration of 50 nM siRNA. Three ul of Pepmute reagent were then mixed by pipetting up and down, incubated 15 minutes at RT, and dropped onto the cultured cells. After 72 h, cells were harvested or fixed for further experiments.

Adenovirus-p35 and empty vector (EV) were made and infected according to the methods of previous study [2, 18].

### Glomerular Isolation

C57BL/6J mice kidneys were drawn from the breeding adult mouse and Glomeruli were isolated by following the method of graded sieving [19].

### Western blot analysis

Cells were harvested by scraping cells and lysed in ice-cold lysis buffer (20 mM Tris, pH 7.5, 150 mM NaCl, 1 mM EDTA, 1 mM EGTA, 1% Triton X-100, 1 mM β-glycerol phosphate, and 1 mM NaF, supplemented with a mixture of protease inhibitor cocktail and 1 mM PMSF), and incubated for 30 minutes on ice. After centrifugation for 20 minutes at 13,000× g at 4°C, the protein concentrations of the supernatants were determined using the BCA protein assay (Pierce, Rockford, Illinois). An equal amount of total protein (20 μg of protein/lane) was resolved on a 4–20%, a 15% or 8% SDS-polyacrylamide gel and blotted onto a PVDF membrane. The membrane was incubated in blocking buffer containing 20 mM Tris-HCl (pH 7.4), 150 mM NaCl, and 0.1% (v/v) Tween 20 (TTBS) plus 5% dry milk (w/v) for 1 h at room temperature, and incubated with primary antibodies overnight at 4°C. The membranes were then washed four times in TTBS, followed by incubation in goat anti-mouse or goat anti-rabbit IgG (H+L)-HRP conjugated secondary antibodies (Amersham Biosciences, 1: 2500) for 2 h at room temperature. Western blots were analyzed using the Enhanced Chemiluminescence (ECL) kit (Pierce) following the manufacturer's instructions.

### Cdk5 kinase assays in vitro

Kinase assays were performed as previously described [20]. Cdk5 was immunoprecipitated from supernatants of lysed cells with the polyclonal C-8 antibody overnight at 4°C and immunoglobulin was isolated using Protein A-sepharose beads for 2 h at 4°C. Immunoprecipitates were washed three times with lysis buffer and then once with 1× kinase assay buffer containing 20 mM Tris-Cl pH 7.4, 1 mM EDTA, 1 mM EGTA, 10 mM MgCl2, 10 μM sodium fluoride and 1 μM sodium orthovanadate. Kinase assays were performed in the same buffer containing 1 mM DTT, 0.1 mM ATP and 0.185 MBq [γ-32P] ATP with 20 μg of histone H1 as the substrate. Histone H1 phosphorylation was performed in a final volume of 50 μl, incubated at 30°C for 60 minutes and stopped by addition of 10% SDS sample buffer and heated at 95°C for 5 minutes. Samples were separated by SDS-PAGE, gels were stained with Coomassie, destained, dried and exposed for autoradiography.

### Immunocytochemistry

Podocytes were cultured and infected on glass cover slips. They were washed twice in PBS, fixed for 30 minutes at room temperature in 4% paraformaldehyde in PBS, and permeabilized with a buffer (25 mM Tris, pH7.4, 150 mM NaCl, and 0.1% Triton X-100) for 15 min. The coverslips were incubated overnight at 4°C with primary antibodies. All antibodies were diluted in PBS with 1% Triton-X-100. After three washes in PBS, coverslips were incubated with fluorescein goat anti-mouse IgG, or Texas Red goat anti-rabbit IgG for 1 h at room temperature, followed by three washes with PBS. Fluorescent images were obtained with a Zeiss LSM-510 laser-scanning confocal microscope and images were managed with Adobe Photoshop.

### In situ cell death detection (TUNEL and cleaved caspase-3 staining assays)

Podocytes were cultured and tranfected with siRNAs using Pepmute siRNA trasfection reagent (SignaGen Laboratory). TUNEL staining was performed according to the manufacturer’s instructions using the in situ cell death detection kit TMR red (Roche). TUNEL staining and cleaved caspase-3 fluorescent images were captured with a Zeiss LSM-510 laser-scanning confocal microscope and images were managed with Adobe Photoshop. Cell counts were performed to measure cell apoptosis.

### Viability of cells assay

Podocytes were cultured and tranfected with siRNAs using Pepmute siRNA trasfection reagent. Viable cells was counted using cell counting kit-8 (CCK-8) according to the manufacturer’s instructions.

### Statistical analysis

Analysis of variance (ANOVA) with Tukey-Kramer adjustment for multiple comparisons was applied. A p-value below 0.05 was considered significant.

## Results

### The expression of Cdk5, p35, and Cdk5 activity in immortalized mouse podocytes and glomeruli

**Fig 1:**
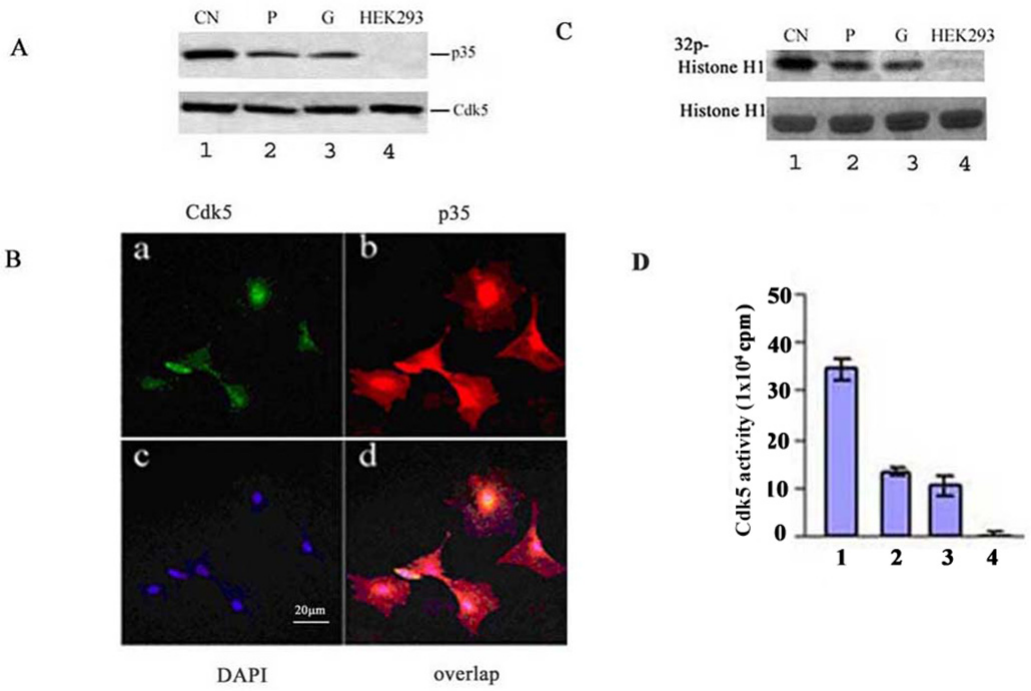
The expression of Cdk5, p35, and Cdk5 activities in podocytes and mouse glomeruli. A. Western blotting analysis showed levels of expressions of p35 and Cdk5. B. Confocal images illustrate the expressions of p35 and Cdk5 in mouse podocytes using immunofluoresence analysis. a: Cdk5; b: p35; c: nuclei; d: overlapped Cdk5 and p35. The scale bar represents 20 μm. C. Cdk5 activity in podocytes and glomeruli. Cdk5 was immunoprecipitated using C-8 antibody from equal amounts of lysates. Immunoprecipitates were then subjected to in vitro histone H1 kinase assays. Autoradiography showed the level of phosphorylated histone H1 in the gels to reflect the Cdk5 activity. The Cdk5 activity of cortical neurons is shown in lane 1; the Cdk5 activity in mouse podocytes and glomeruli in lanes 2 and 3; a negative Cdk5 activity in HEK293 cells shown in lane 4. D. The bar graph represents mean optical density measurements of phospho-histone (32P-histone H1) in autoradiographs. Data are expressed as means of three separate experiments.

Mouse cortical neuronal cells expressing Cdk5 and p35, were used as positive control [1]. The expression of Cdk5 was approximately equal in mouse cortical neurons (CN), cultured immortalized mouse podocytes (P), mouse glomeruli (G), and HEK293 cells (Fig 1A. lower panel, lane 1, 2, 3 and 4, respectively). p35, however, was positively expressed in mouse podocytes and glomeruli (Fig 1A. upper panel, lane 2&3), and at higher levels in the cortical neurons (Fig 1A. upper panel, lane 1), but absent in HEK293 cells (Fig 1A. upper panel, lane 4). We further confirmed the expression of Cdk5 and p35 in immortalized cultured podocytes by using immunofluorescent analysis (Fig 1Ba and 1Bb, respectively); Cdk5 and p35 localization overlapped in the podocytes (Fig 1Bd). Cdk5/p35 activity assays in podocytes and glomeruli also showed activities (Fig 1C & 1D. lane2&3), that were much lower than in cortical neurons (compared lane 2&3 with lane 1). There was no Cdk5 activity in the HEK293 cells (Fig 1C & 1D. lane4). These results indicate that p35 is present in podocytes, activates Cdk5 activity and may play an important role in podocyte regulation.

### Time course expression of Cdk5 and p35 in podocyte cultures and cortexes from developing mouse kidneys

**Fig 2:**
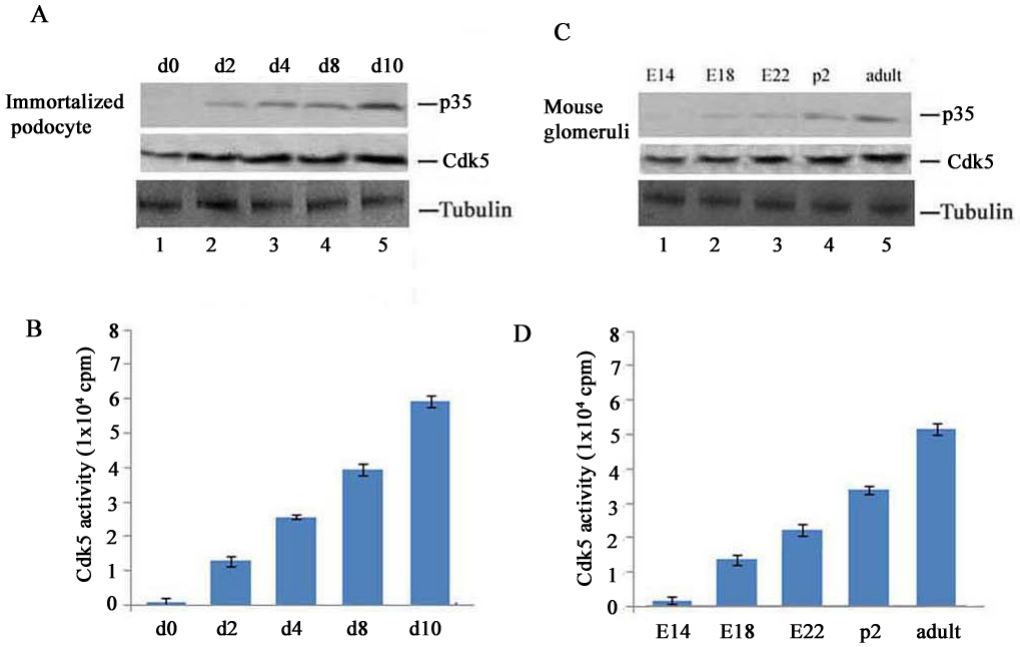
The expression of Cdk5/p35 in the immortalized mouse podocytes and the cortexes of mouse kidneys over time. A. Western blotting analysis showed the p35 and Cdk5 expression levels in the cultured mouse podocytes at 0 day (d0: lane1), 2 days (d2: lane 2), 4 days (d4: lane 3), 8 days (d8: lane 4) and 10 days (d10: lane 5). B. The bar graph illustrates an increasing Cdk5 activity in the cultured mouse podocytes from day 0 to day 10. C. Western blotting showed the p35 and Cdk5 expression levels in the cortexes from embryos to the adult: E14 (lane 1), E18 (lane 2), E22 (lane 3), p2 (lane 4) and adult (lane 5) mouse kidneys. D. bar graph showed an increasing Cdk5 activity levels in the cortexes of mouse kidneys during the above time (E14-adult). Data are expressed as means of three separate experiments.

To detect the expression levels of Cdk5 and p35 during podocyte maturation in culture and during kidney development we used Western blots to determine the expression of Cdk5 and p35 in cultured podocytes and cortexes from mouse kidneys at different times (Fig 2A & 2C). The expression of Cdk5 tended to increase over time in cultured podocytes (Fig 2A. lower panel, lane 1–5) and kidney cortexes (Fig 2C. lower panel, lane 1–5). p35 expression, negative in podocytes on day zero and E14 kidney cortexes (Fig 2A & 2C. up panel, lane 1), increased in cultured podocytes (Fig 2A. up panel, lane 2–5) and kidney cortexes (Fig 2C. up panel, lane 2–5) reaching highs on day-10 podocytes (Fig 2A. up panel, lane 5) and adult kidney cortexes (Fig 2C. up panel, lane 5). Cdk5 activities increased in a similar manner in podocytes and kidney cortexes (Fig 2B & 2D, bar graphs). These results further suggest that Cdk5/p35 activity plays an important role in podocyte development.

### Over expression of p35 promotes podocyte differentiation

**Fig 3:**
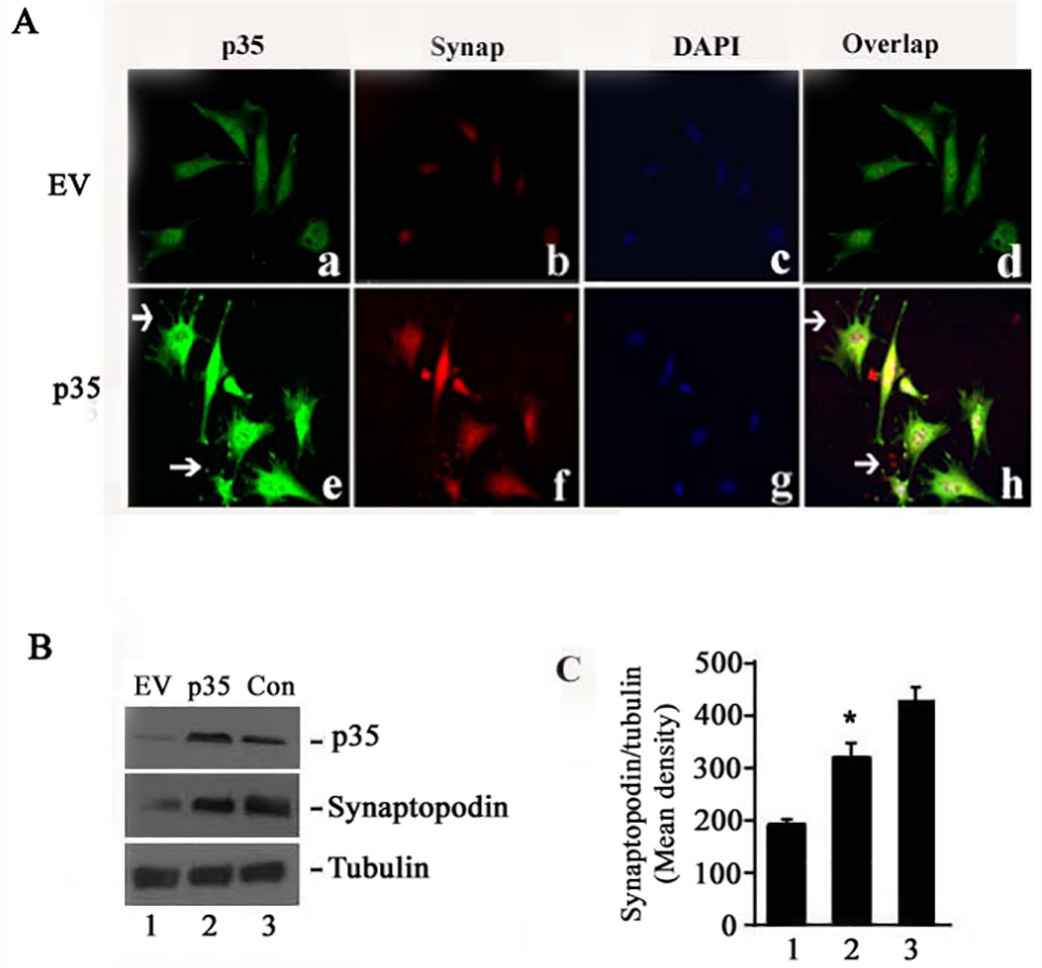
Transfection of p35 induces podocyte differentiation. The p35 gene and empty vector (EV) were infected into cultured podocyte (day 1). After 48h incubation, cells were fixed or harvested for further detection of p35 and synaptopodin by immunofluorescence and western blots. A. Immunofluorescence show that p35 gene infected podocytes sucessfully upregulated p35 expression and increased podocyte spike like differentiate processes (e vs a), and also raised synaptopodin expression (f vs b). B. Western blots display that raised synaptopodin level in p35 gene transfected podocytes. Cultured mature podocytes(day 10) was used as control(con). C. A bar graph illustrates increased synaptopodin expression in p35 gene transfected podocytes(bar 2 vs bar 1), p<0.01.

To investigate the relation between Cdk5/p35 and podocyte differentiation, we over expressed p35 gene in podocytes and observed cell differentiation. Podocytes were infected with adenovirus—p35 and EV on the day1of differentiation phase culture (37°C without γ-IFN). After 48h, cells were fixed or harvested for ICC staining or WB analysis. The results showed that p35 infected cells exhibit obviously spike like differentiation processes (Fig 3Ae–3Ah, arrows). However, there are negative spike like processes in EV infection cells (Fig 3Aa–3Ad). The expression of synaptopodin, a podocyte differentiation marker, was increased in p35 infected cells (Fig 3f vs 3b). This result indicated that p35 may promote the podocyte differentiation. To confirm the result, we detected the expression of synaptopodin with WB using the same cells as above. The result showed that synaptopodin expression in the p35 infected cells was remarkably increased (Fig 3B, middle panel, lane 2, comparing to lane 1, p<0.05); lane 3 is a positive control (10days cells cultured in differentiate phase). Above results indicates that Cdk5/p35 plays an important role in podocyte differentiation.

### Knockdown of Cdk5 expression induced apoptosis, decreased the expression of WT1 and viable cell number in immortalized mouse podocytes

**Fig 4:**
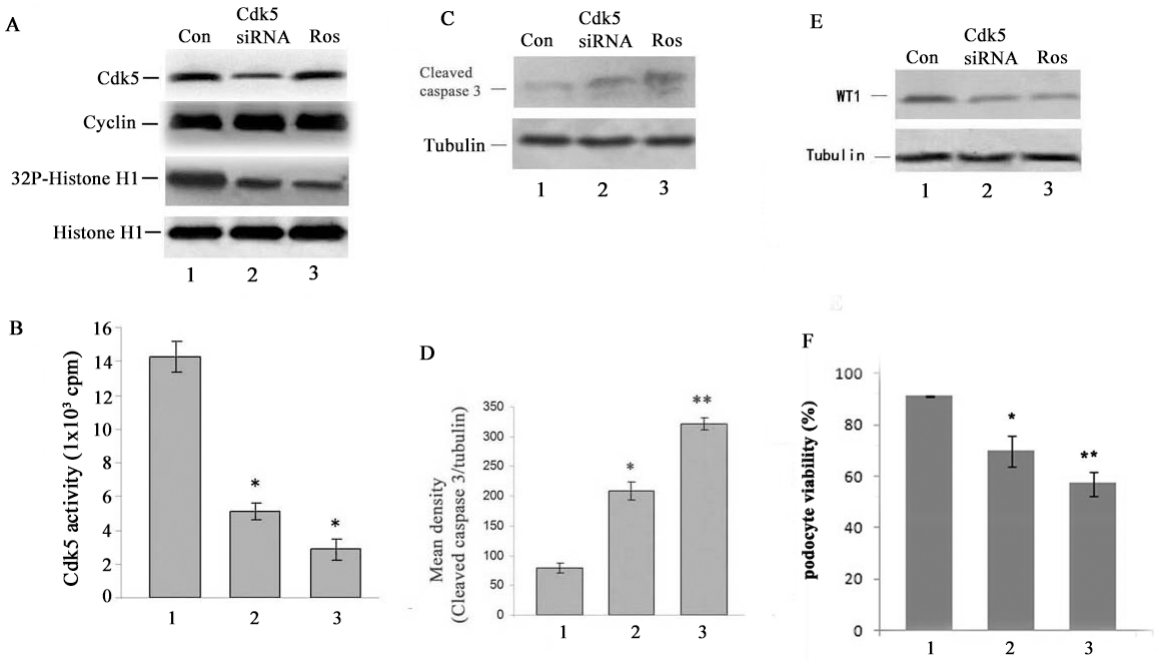
Cdk5 siRNA knockdown decreased Cdk5 activity, diminished WT1 expression and viable cell numbers, and increased cleaved caspase 3 level. The Cdk5 siRNA and control siRNA were transfected into cultured podocyte (day 7), and roscovitine was used as a positive control to treat the cells. After 72h incubation, cells were harvested and submitted to the Western blotting and kinase assay analysis. A&B. Western blots show that Cdk5 siRNA-transfected podocytes successfully decrease Cdk5 expression (upper panel, lane 2) and activity (3rd panel, lane 2 and bar 2, compared to the control siRNA cells, p<0.05), while the expression of cyclin I remain unchanged (2^nd^ panel). C&D. Cdk5 siRNA induced cleaved caspase 3 expression (upper panel, lane 2; bar 2 vs bar 1, p<0.05). E. Western blotting analysis showed a decline of WT1 expression in Cdk5 siRNA podocytes (upper panel, lane 2). F. Cdk5 siRNA treated podocytes showed a decline of cell viability compared to control siRNA transfected podocytes, p<0.05.

It should be noted that Wilms’ Tumor 1(WT1), a transcription factor, is required for podocyte maturation and is often used as a molecular marker for differentiated podocytes [21–22]. To further confirm the effect of Cdk5 in podocytes, we deleted Cdk5 from cultured mouse podocytes by using Cdk5 siRNA transfection technology. The Cdk5-siRNA transfected into cultured podocytes (day 7). After 72h incubation, cells were harvested for western blotting, kinase assay and viable cell counting. Cdk5 expression in the Cdk5 siRNA cells is significantly eliminated (Fig 4A. upper panel, line 2) without affecting cyclin I expression (Fig 4A. 2^nd^ panel, lane 2). And a decreased Cdk5 activities (Fig 4A. 3rd panel, line 2; and Fig 4B. bar graph, bar 2 vs bar 1, p<0.01). The roscovitine was used as positive control (Fig 4A. lane3 and 4B, bar 3). To test if the down regulation of Cdk5 affected podocyte survival, apoptosis analysis was carried out by western blotting using cleaved caspase3 antibodies, a cell apoptosis marker. The results showed that the cleaved caspase3 expression was significantly upregulated in the Cdk5 siRNA treated cells (Fig 4C. lane 2 and Fig 4D, bar 2), compared to the control siRNA cells (p<0.01). This was confirmed by a reduction in WT1, a podocyte-specific marker (Fig 4E. lane 2) with decreased cell number (Fig 4F, bar 2), compared to the control siRNA cells (p<0.05).As a positive control, roscovitine-treated cells showed a significantly increasing level of cleaved caspase 3 (Fig 4C. lane 3), reduction in WT1 expression (Fig 4E. lane 3) and viable cell number (Fig 4F, bar 3). These results show that knockdown of the Cdk5 expression and activity caused cell apoptosis and decreased podocyte number.

### Knockdown of p35 decreased the expression of WT1 and viable cell number without affecting cleaved caspase 3 apoptosis marker as detected by western blotting

**Fig 5:**
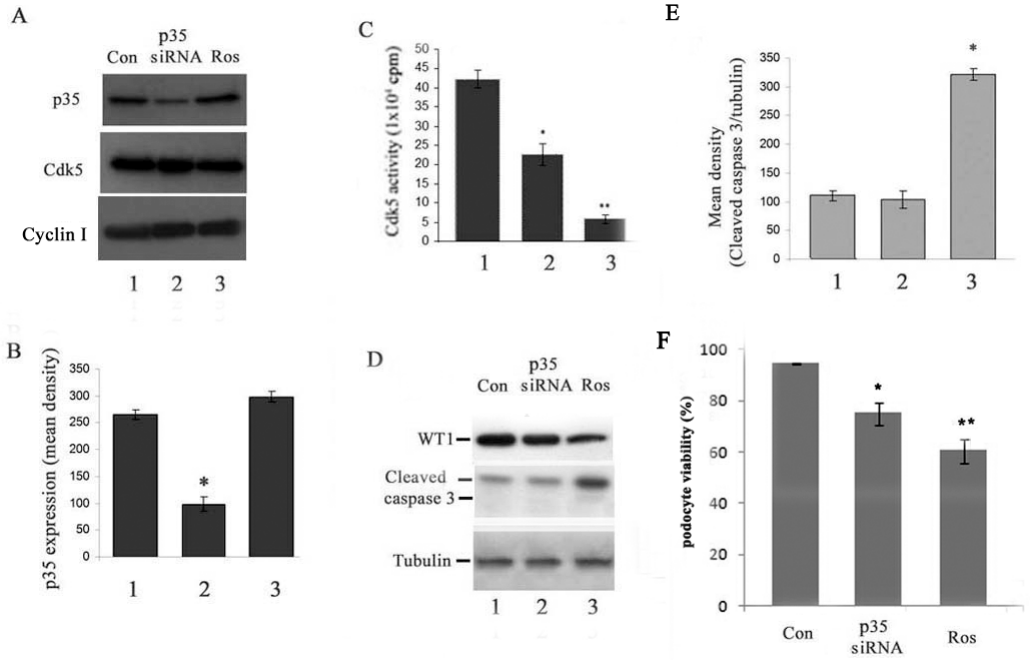
p35 siRNA knockdown decreased Cdk5 activity, diminished WT1 expression and viable cell numbers, but did not affect a cell apoptosis marker as detected by WB. The p35 siRNA and control siRNA were transfected into cultured podocyte (day 7), and roscovitine was used as a positive control to treat the cells. After 72h incubation, cells were harvested and submitted to the Western blotting and kinase assay analysis. A&B. Western blots show that p35 siRNA-transfected podocytes successfully decrease p35 expression (upper panel, lane 2) compared to the control siRNA cells (p<0.05), but did not affect cyclin I expression (3^rd^ panel). C. p35 siRNA reduced Cdk5 activity. D. Western blotting analysis showed a decline of WT1 expression in p35 siRNA podocytes (upper panel, lane 2 compared to lane 1, p = 0,048), but did not affect cleaved caspase 3 expression (middle panel, lane 2 compare with lane 1). E. A bar graph showed there was no significant difference of cleaved caspase 3 expression between p35 siRNA and control siRNA treated cells, but roscovitine treated cells showed robust increase of cleaved caspase 3. F. p35 siRNA treated podocytes showed a decline of cell viability compared to control siRNA transfected podocytes, p<0.05.

P35 is the main activator of Cdk5. We now ask whether knockdown of p35 with p35 siRNA results in decreased cell number and elevated cleaved caspase 3 (Fig 5). The results showed a down regulation of p35 expression in Fig 5A, lane 2. There is a significant difference compared to the control siRNA treated cells (Fig 5B. bar graph, bar 2 vs bar 1, *P*<0.01). There is also a significant decrease in Cdk5 activity in p35 siRNA and roscovitine treated podocytes (Fig 5C. bar 2 and 3 vs bar 1, p<0.01 and <0.001, respectively). Cyclin I expression, however, was not changed in the three groups of cells (Fig 5A, panel 3). Moreover, WT1 expression and viable cell number decreased in p35 siRNA treated cells (Fig 5D. upper panel, lane 2, compared to the lane 1, p = 0.048; and Fig 5F. bar 2, compared to the lane 1, p<0.05, respectively). However, the cleaved caspase-3 marker was unaffected by using western blotting; there was no significant difference in the p35 siRNA treated cells (Fig 5D, middle panel, lane 2, 5E, bar graph, bar 2 vs bar 1, p>0.05). Positive control showed the same results as in Fig 4.

### Knockdown of p35 resulted in defective podocyte morphology and apoptosis

**Fig 6:**
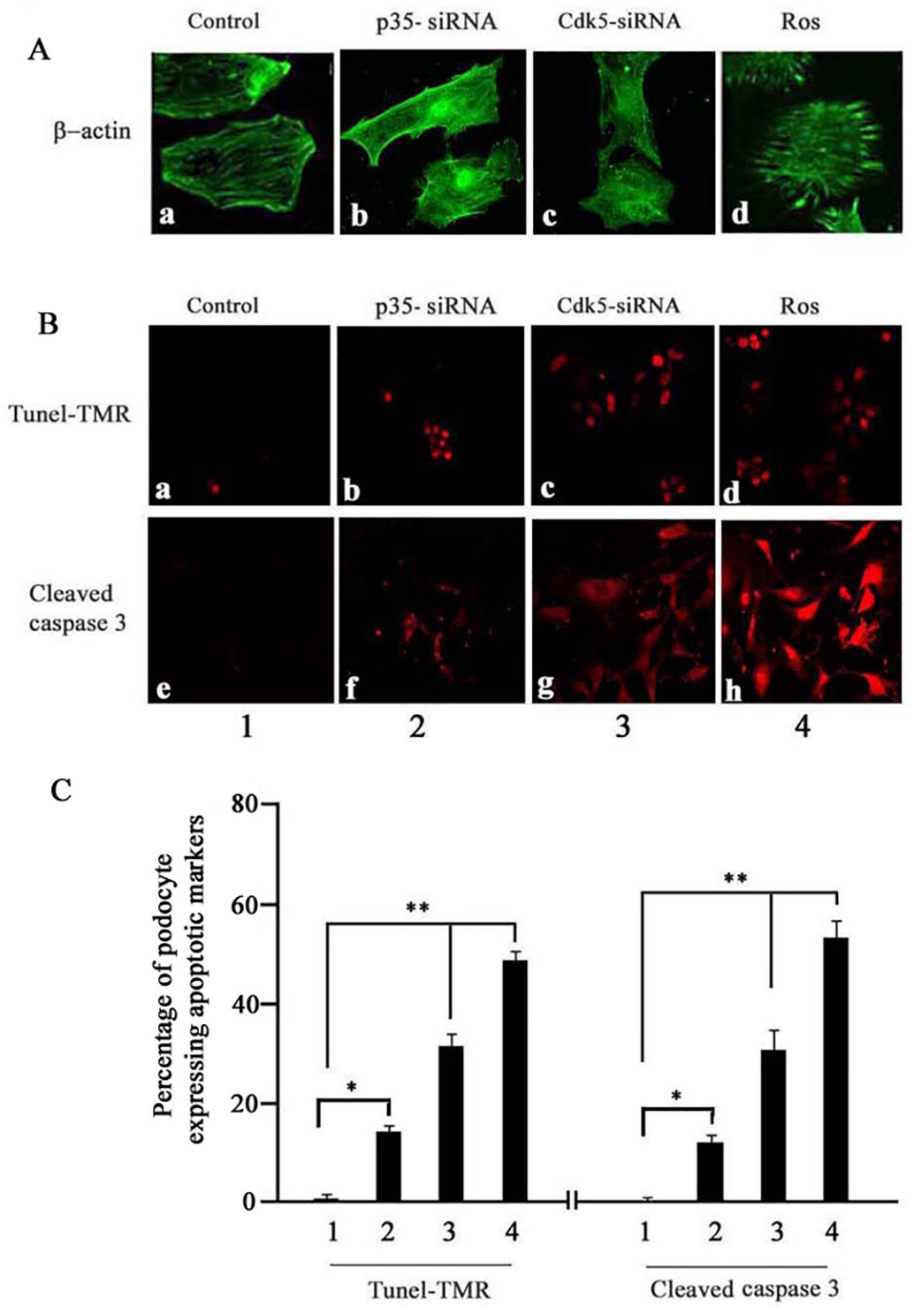
p35 siRNA directly induced podocyte apoptosis and abnormal morphology as detected by TUNEL analysis and immunofluorescence staining respectively. After p35 siRNA transfection 72 hs, cells were fixed and submitted to TUNEL analysis and immufluorescence staining using cleaved caspase 3 and β-actin antibodies. A. podocyte morphological analysis (a: control siRNA; b: p35 siRNA; c: Cdk5 siRNA; d: roscovitine); B. podocytes apoptosis analysis: upper panel: tunel analysis (a: control siRNA; b: p35 siRNA; c: Cdk5 siRNA; d: roscovitine); lower panel: cleaved caspase 3 staining (e: control siRNA; f: p35 siRNA; g: Cdk5 siRNA; h: roscovitine). C. TUNEL staining and cleaved caspase-3 fluorescent images semi-quantity anlysis. The images were captured with a Zeiss LSM-510 laser-scanning confocal microscope and were managed with Adobe Photoshop. Cell counts were performed as follows: 10 independent fields were analyzed with a total of 500 cells where TUNEL and cleaved caspase-3 could be counted. DAPI staining gave the total number of podocyte cells. The bar graph shows the quantity of podocyte apoptosis expressed as mean from three separate transfections.

Brinkkoetter et al had shown that deletion of p35 in podocyte or p35 KO in mice kidneys didn’t directly induce podocyte apoptosis, but did so indirectly [5]. Our results confirmed their data although, we showed that podocyte number decreased in the p35 siRNA treated cells. To further explore whether depletion of p35 affects podocyte survival, we examined podocyte morphology by immunocytochemical staining with β-actin antibody. The results showed that podocyte structure was abnormal in p35 siRNA transfected cells (Fig 6Ab) similar to Cdk5 siRNA transfected cells (Fig 6Ac), which showed the cell morphological changes and actin rearrangement with a loss of the typcal transverse stress fibers; whereas roscovitine destroyed podocyte structure (Fig 6Ad). The results suggest that p35 knockdown may indeed cause podocyte apoptosis. Accordingly using tunel analysis we could demonstrate significant apoptosis in the p35 siRNA treated cells (Fig 6Bb and 6C. TUNEL -TMR, bar 2 vs bar 1 p = 0.041,). To further confirm this result, the same experiment was carried out using immunofluorecence staining with cleaved caspase 3 antibody. A similar result was obtained (Fig 6Bf and 6C. cleaved caspase 3, bar 2 vs bar 1, p = 0.036). The positive control cells which were treated with Cdk5 siRNA and roscovitine showed remarkable apoptosis in both staining analysis (Fig 6Bc, 6Bd, 6Bg and 6Bh). The significant differences were shown in Fig 6C (both TUNEL -TMR and cleaved caspase 3, bar3 and 4 vs bar 1, P<0.0001). Evidently, TUNEL and immuflouresence staining are more sensitive than Western blotting as a measure of apoptosis. All these findings indicated that Cdk5/p35 expression and activities play a cytoprotective role in maintaining podocyte function and survival.

## Discussion

Podocytes are terminally differentiated glomerular epithelial cells which adhere to the outer flank of the glomerular basement membrane, that play an essential role in the prevention of proteinuria. Similar to other terminally differentiated cells podocytes have a limited capacity to proliferate. Accordingly, when they undergo apoptosis, overall cell number tends to decrease. Loss of podocytes is involved in the onset and progression of many primary or secondary kidney diseases [23–25].Dysfunction and loss of podocytes has become a critical indicator of the severity of glomerular damage and sclerosis. Cdk5 is activated by p35, p39, and cyclin I, and plays multiple roles in the growth and function of such terminally differentiated cells as neurons, pancreatic β cells and podocytes [17–18,26–27]. A recent study showing a role for Cdk5/p35 in the pathogenesis of high glucose induced podocyte injury [28].

Griffin et al showed that Cdk5 protein increased during differentiation of cultured podocytes, and that p35 is also expressed in adult mouse glomeruli and cultured podocytes[5,17].Here, we first confirmed the expression of Cdk5 and p35 in immortalized cultured mouse podocytes and glomeruli, and also showed that expression of both proteins increased over time in culture and during glomerular development respectively. This is the first demonstration that cdk5 and p35 are expressed in differentiating embryonal glomeruli, and persist in mature adult glomeruli. Similarly, in cultured podocytes, both Cdk5 and p35 expressions increased during the switch from a proliferating phenotype to a differentiated and quiescent one. These results suggest that Cdk5/p35 expression is important in podocyte differentiation. These results were confirmed by over-expressing p35 in podocytes which showed a spike like differentiation processes and increased expression of synaptopodin, a podocyte differentiation marker, in the beginning of differentiation culture condition.

Podocytes and neurons share many similarities, both cells are specialized, terminally differentiated cells, with a complicated cytoarchitecture and wide, highly organized cell processes [29]. It was originally thought that p35, an activator of Cdk5, is largely expressed in the neurons responsible for high levels of Cdk5 activity [30]. The activity is essential for neuronal migration and ordered cortical lamination during development of the mammalian brain [31]. Cdk5 knockout (KO) mice exhibit perinatal lethality and defective positioning of several types of neurons [15], p35 KO mice, however, show a milder phenotype of abnormal neuronal cytoarchitecture [15–16]. We ask whether a Cdk5 KO and /or a p35 KO express an abnormal podocyte or glomerular phenotype. Our current study showed that a Cdk5 KO (siRNA-induced) resulted in podocyte apoptosis, decreased WT1 and diminished podocyte number. These results, consistent with Taniguchi’s reports [32] indicate a crucial role of Cdk5 in podocyte survival.

On the other hand, deletion of p35 in podocytes cultures or in p35 KO mouse kidneys did not induce podocyte apoptosis and loss directly, but affected them indirectly [5, 21]. We obtained a similar result; the cleaved caspase 3 marker of apoptosis showed no effect in the p35 KO (siRNA-induced) podocytes but WT1 and viable cell number decreased indicating loss of podocyte number. Because of this finding, we further applied TUNEL and immufluorescence staining for apoptosis analysis in the p35 knockdown cells. Here, knockdown of p35 evoked abnormal podocyte morphology due to actin rearrangement (Fig 6Ab), and podocyte apoptosis (Fig 6Bb & 6Bf). This finding indicates that p35 is also involved in maintaining podocyte normal morphology and function. It also indicates that the TUNEL and immunofluorescence staining maybe more sensitive than WB for cell apoptosis analysis. The mechanism of p35 KO induction of podocyte apoptosis is not well understood. Apoptosis is differentiated from necrosis by morphologic and functional features and by the requirement for energy and intracellular proapoptotic proteins [33]. It is likely that decreased Cdk5 activity-dependent apoptosis results from deregulation of podocyte proliferation and differentiation. Cdk5 is critical for the cell development and differentiation in various cell types, such as myoblasts [34], lens epithelial cells [35], and human hematopoietic cells [17, 36]. Maintaining podocyte differentiation is a prerequisite for normal podocyte cytoskeletal structure and survival. Since, cycline I is another activator of cdk5 in podocytes[5, 17], it is likely that decreased p35 causes cell apoptosis indirectly. Our study showed that knockdown either Cdk5 or p35 does not affect cycline I expression. This result indicates that knockdown p35 caused podocytes apoptosis is independent from cycline I. A recent study examined the relationship between subcellular distribution of Cdk5 with its activator cyclin I and p35 in podocytes and found that the predominance of cyclin I mediates the nuclear localization of Cdk5, whereas the predominance of p35 results in a membranous localization of Cdk5. These results strongly indicate that cyclin I and p35 do not compensate for one another and that both are needed to maximize cell survival [27]. The presence of p39 in the podocyte has not been determined.

An alternative mechanism for the role of p35 in survival is seen in a study of a mutant p35 in Drosophila [37]. The mutation causes an increase in apoptotic and necrotic cell death, axonal fragmentation, accumulation of autophagosomes packed with crystalline-like depositions, and finally leads to neurodegeneration. The study revealed aggregated depositions of endogenous material as well as enhanced accumulation of autophagic organelles from ultrastructural analysis of the p35 mutant, which may contribute to neuronal death [37]. Whether it occurs in a podocyte p35KO and initiates apoptosis will be an important subject for future investigation.

In summary, in this study we show that both Cdk5 and p35 are expressed in cultured immortalized mouse podocytes and glomeruli and increase in expression over time. Knockdown of Cdk5 or p35 caused podocyte apoptosis and morphological abnormalities. Taken together, these data demonstrate that Cdk5/p35 expression and activity are essential for podocyte survival.
